# Competitive species interactions constrain abiotic adaptation in a bacterial soil community

**DOI:** 10.1002/evl3.83

**Published:** 2018-09-25

**Authors:** James P. J. Hall, Ellie Harrison, Michael A. Brockhurst

**Affiliations:** ^1^ Department of Animal and Plant Sciences University of Sheffield Western Bank Sheffield S10 2TN United Kingdom; ^2^ Department of Biology University of York Wentworth Way York YO10 5DD United Kingdom

**Keywords:** Adaptation, competition, experimental evolution, nutrient scavenging, *Pseudomonas fluorescens*, soil microbiology

## Abstract

Studies of abiotic adaptation often consider single species in isolation, yet natural communities contain many coexisting species which could limit or promote abiotic adaptation. Here we show, using soil bacterial communities, that evolving in the presence of a competitor constrained abiotic adaptation. Specifically, *Pseudomonas fluorescens* evolved alone was fitter than *P. fluorescens* evolved alongside *Pseudomonas putida*, when *P. putida* was absent. Genome analyses indicated this was due to mutation of the acetate scavenger *actP*, which occurred exclusively, and almost universally, in single‐species‐evolved clones. *actP* disruption was associated with increased growth in soil compared with wild‐type *actP*, but this benefit was abolished when *P. putida* was present, suggesting a role for carbon scavenging transporters in species interactions, possibly through nutrient competition. Our results show that competitive species interactions can limit the evolutionary response to abiotic selection, because the fitness benefits of abiotic adaptive mutations were negated in more complex communities.

**Impact summary**

Organisms evolve in response to their physical environment. This “abiotic” adaptation can, however, be influenced by the presence of other evolving species, which could inhibit or enhance evolution. We tested this experimentally by evolving populations of the bacterium *Pseudomonas fluorescens* in soil, either on their own or alongside another species of soil bacterium, *Pseudomonas putida*. We found that the presence of *P. putida* inhibited abiotic adaptation to the soil environment. *Pseudomonas fluorescens* that had evolved alone had higher fitness in soil than *P. fluorescens* that had evolved alongside *P. putida*. By analyzing the genomes of evolved *P. fluorescens*, we identified frequent mutations in the gene *actP*, encoding an acetate‐scavenging transporter, but only in clones that had evolved alone. Bacteria with mutated *actP* grew faster, but only when *P. putida* was absent. Together, our work shows that species interactions can limit abiotic adaptation and provides a rare glimpse of the underlying genetics.

Although most evolutionary theory considers single species evolving in isolation, virtually all organisms live in diverse, complex communities. Competitive species interactions are ubiquitous in ecological communities, yet the effect of interspecific competition on evolutionary adaptation to the abiotic environment remains unclear. Evolutionary studies of both natural and experimental communities suggest that interspecific competition could either limit or promote abiotic adaptation, depending upon its ecological effect on the focal species (Collins 2011; Barraclough 2015). Abiotic adaptation could be limited by interspecific competition if competitor species weaken the focal species’ response to abiotic selection. This could occur if, in the focal species, competition reduces population size and, consequently, the genetic variation available to abiotic selection (Lanfear et al. 2014), or if biotic and abiotic adaptations are subject to fitness trade‐offs or negative genetic correlations (Scanlan et al. 2015). Alternatively, competitor species could alter the ecological opportunities afforded to the focal species by the abiotic environment. This could limit adaptation if competitor species fill ecological niches faster than the focal species can adapt to them (i.e., species sorting) (de Mazancourt et al. 2008), or promote adaptation if competition for its current ecological niche drives the focal species to adapt to alternative ecological niches (i.e., character displacement) (Grant and Grant 2006; Zhang et al. 2012; Stuart et al. 2014; Jousset et al. 2016), or if the competitor species create new ecological niches for the focal species to exploit (e.g., through facilitation or cross‐feeding) (Harcombe 2010; Lawrence et al. 2012). Although there is evidence from a variety of systems that interspecific competition frequently alters the trajectory of abiotic adaptation, very few studies have identified the genetic basis of adaptive traits differentially selected in the presence versus absence of competing species (Jones et al. 2012; Lamichhaney et al. 2015), limiting our mechanistic understanding of evolution in competitive communities.

Experimental evolution allows the effect of competitor species on abiotic adaptation of a focal species to be directly tested, although to date there have been few studies of this kind. A key finding of experimental evolution studies where microbes evolve alone in simple, unstructured environments has been their rapid and continued genotypic and phenotypic adaptation to their abiotic environment (Wiser et al. 2013). However, this pattern can be altered by the presence of competitors. Addition of competitor algal strains constrained abiotic adaptation of focal algal strains to high CO_2_, apparently due to a trade‐off between competitive ability and evolvability (Collins 2011). Similarly, the degree of adaptation of the bacterium *Pseudomonas fluorescens* observed when evolving in the presence of competing strains was negatively associated with their competitiveness due to greater reductions in the population size of the focal species (Zhao et al. 2018). Conversely, the presence of competitor species promoted the evolutionary diversification of morphological (Zhang et al. 2012) and metabolic (Jousset et al. 2016) traits of *P. fluorescens*, suggesting that interspecific competition drove the evolution of character displacement to exploit vacant ecological niches. In more complex five‐species bacterial communities, initially competitive interactions evolved toward increased facilitation as coexisting species evolved to metabolize their neighboring species’ waste products (Lawrence et al. 2012). This led to higher community productivity, at the expense of reduced growth in monoculture, suggesting that while competitor species created new ecological niches, adapting to these niches limited adaptation to the abiotic environment per se. Here, we extend these previous studies to understand the underlying genetic basis of abiotic adaptation in the presence versus absence of competitor species.

We experimentally evolved *P. fluorescens* SBW25 in soil microcosms for ∼440 generations (Hall et al. 2016, 2017). Our experiment was originally established to investigate the evolution and population dynamics of bacteria harboring a mercury resistance (Hg^R^) plasmid under different ecological conditions: in single species or coculture with *Pseudomonas putida* KT2440, and where the plasmid was net costly (i.e., in the absence of mercury) or beneficial (i.e., in the presence of mercury). Previously, we reported the effects of these treatments on plasmid dynamics (Hall et al. 2016) and on gene mobilization (Hall et al. 2017). Here, we quantify changes in the competitive fitness of *P. fluorescens* relative to its ancestor to estimate the degree of abiotic adaptation to the soil environment that occurred in the different treatments. We then use genome analysis of evolved *P. fluorescens* clones to identify genetic loci associated with abiotic adaptation and perform growth experiments to test their phenotypic effects. We report that interspecific competition constrained abiotic adaptation of *P. fluorescens* because the fitness benefits of abiotic adaptive mutations were negated in a more complex community.

## Materials and Methods

### BACTERIAL CULTURE

For the evolution experiment, *P. fluorescens* SBW25 and *P. putida* KT2440 were grown in soil microcosms in a full‐factorial design with two levels of Hg(II) (0 μg/g and 16 μg/g), two levels of culturing (single species and coculture), and two levels of starting plasmid status (50% pQBR57 carriers, or no plasmid, as a control to identify evolution unrelated to the plasmid) (Fig. S1). Six populations were established for each combination of treatments, half with a gentamicin‐resistance (Gm^R^) marker and half with streptomycin resistance and lacZ markers (Sm^R^‐*lacZ*), to control for the effects of the marker genes on evolution. Populations were cultured for ∼440 generations (65 transfers) before clones were isolated for assay. Full details of methods can be found in Hall et al. (2016, 2017). Here, we focus on the effect of single species versus coculture in the plasmid‐containing treatment, but the mercury treatment and the plasmid‐free control treatment are included in some analyses because these were part of the evolution experiment. However, as is described in Results section, our main findings were found to be independent of these aspects.

For competition assays, six or seven plasmid‐bearing *P. fluorescens* clones were picked at random from each plasmid‐containing population at the final timepoint and reisolated on KB agar with 20 μM Hg(II) to ensure plasmid carriage. Note that one Gm^R^ cocultured population in 0 μg/g Hg(II) lost the plasmid, and therefore fitness was not measured for this population. Cultures grown overnight in KB broth were pelleted, diluted 10‐fold in M9 salt buffer, mixed in a ∼50:50 ratio with ancestral plasmid‐bearing competitors, and 100 μL used to inoculate 10 g soil microcosms, which had been preconditioned with 1 mL H_2_O containing either 0 μg/g Hg(II) or 16 μg/g Hg(II). Evolved Gm^R^ clones were competed against Sm^R^‐*lacZ* ancestors, whereas evolved Sm^R^‐*lacZ* clones were competed against ancestral Gm^R^ clones. Starting samples were diluted and spread on KB agar supplemented with X‐Gal to estimate counts for each genotype. After 4 days growth in the soil microcosm, bacteria were extracted by soil wash (Hall et al. 2016) and spread on KB + X‐gal to enumerate cfu/g soil. Fitness of each competing Gm^R^ strain was calculated as the difference in Malthusian parameters,s Gm =(loge(Gm end Gm start )−loge(Sm end Sm start ))/t to give a coefficient measured (day^–1^). This can be multiplied by generation time (estimated as ∼0.46 days; Hall et al. 2016) to give a coefficient per generation (Chevin 2011) (Table S3). To standardize across markers, we subtracted from each fitness measurement the mean fitness of the ancestral Gm^R^‐labeled strain, and, where the evolved strain was in the Sm^R^‐*lacZ* background, subtracted the resulting value from zero to give *s*_evolved_, the fitness of the evolved strain relative to the ancestor.

For growth assays with and without *P. putida*, *P. fluorescens* overnight cultures were prepared as described above and mixed either with approximately equal numbers of *P. putida* or with sterile M9 buffer. Growth in acetate media was performed by inoculating M9 minimal media supplemented with 1% glycerol or 0.3 mM sodium acetate with approximately 2.5 × 10^6^ bacteria in the exponential phase and allowed to grow for 48 h. Disc diffusion assays were performed by spreading overnight cultures (100 μL of a 1:100 dilution of overnight culture) onto KB agar, with 10 μL of 1% potassium tellurite solution added to a 5‐mm disc of sterile Whatman #1 filter paper in the center. Minimum inhibitory concentration (MIC) assays were performed by subculturing overnight cultures and growing to exponential phase (OD_600_ ∼0.4) and diluting 1:1000 in KB media containing defined concentrations of tellurite.

### SEQUENCE ANALYSIS

Variants were identified from genome resequencing using the GATK HaplotypeCaller (McKenna et al. 2010) and the Bacterial and Archaeal Genome Analyser (Williams et al. 2016) and are described in Hall et al. (2017), and data are available on Dryad (https://doi.org/10.5061/dryad.6gf28). Evolved clones had between two and eight mutations (*P. fluorescens*) or between one and 10 mutations (*P. putida*), with a median of 4 in both species. We could not detect any systematic differences in chromosomal mutations between plasmid‐bearing and plasmid‐free clones isolated from the same population, therefore to avoid issues associated with differential sampling depth in these analyses we used only the plasmid‐containing clone to represent those populations from which more than one clone was sequenced (however, similar results were obtained when all clones were analyzed). To investigate the effects of mercury treatment and coculture on genome evolution of both species, we used the “vegan” package to perform permutational multivariate analysis of variance (MANOVA) (Anderson 2001; Zapala and Schork 2006). For this analysis, we excluded the plasmid‐free treatments due to lack of data from some treatment combinations. A matrix was generated describing, for each population, the presence (1) or absence (0) of a mutation at each locus, from which Euclidean distances between populations were calculated and the betadisper() and adonis() functions were used to test model assumptions, and investigate within‐ versus between‐treatment variance, respectively (Scanlan et al. 2015; Harrison et al. 2017).

Parallel targets of mutation were identified as loci with mutations in more than one population. To investigate specific parallel mutations associated with culture treatment, with mercury treatment, or with the presence of the plasmid, we looked for chromosomal loci which were mutated in at least two populations within one level of the treatment (e.g., single‐species culture) and did not occur in any population within the other level of that treatment (e.g., coculture). This identified 23 candidate genes (Table S1), on which Fisher's exact tests were performed to test for association with each treatment. Each result was compared with a sequential Bonferroni‐corrected alpha to test for significance.

### STATISTICS

We analyzed *s*_evolved_ from competition experiments in a linear mixed‐effects model (LMM) using the R package “nlme”, using culture treatment, evolved mercury conditions, and test mercury conditions as fixed effects, and the population from which a clone was taken as a random effect, to account for repeated measures. Initial data exploration suggested that a heterogeneous variance structure and Box‐Cox transformation were required to meet model assumptions. Using AIC comparisons, we identified that models in which variance varied between populations best fitted the pattern. We initially fitted a “beyond optimal” model including all interactions, and performed likelihood ratio tests on nested models to identify and remove nonsignificant interaction effects. No interaction terms were found to be significant. The significance of remaining fixed effects was estimated by likelihood ratio tests.

To investigate population densities from the evolution experiment, Box‐Cox transformed data were analyzed in an LMM using culture treatment, mercury, plasmid carriage and transfer and their interactions as fixed effects, and population as a random effect on intercept and slope to account for repeated measures. Model selection proceeded as described for competition experiments above.

To test for the effect of mutated *actP* on growth in the presence or absence of *P. putida*, we analyzed the ratio of Malthusian parameters m alone /mco-culture in an LMM using *actP* status as a fixed effect and clone as a random effect. Differences in tellurite inhibition were analyzed by comparing areas under the curve (Bottery et al. 2017), or calculating the zone of inhibition using ImageJ, while growth in minimal acetate was analyzed as m acetate /m glycerol . In each of these cases, data were analyzed in an LMM using *actP* status as a fixed effect and clone as a random effect. Similar results were obtained when analyzing the differences, rather than ratios, of Malthusian parameters.

Analyses were performed using R (R Foundation for Statistical Computing, Vienna, Austria) and plots were created using “ggplot2” (Wickham 2010). Full data and analysis are provided in a package hosted on Figshare (10.15131/shef.data.7043279).

## Results

**Figure 1:**
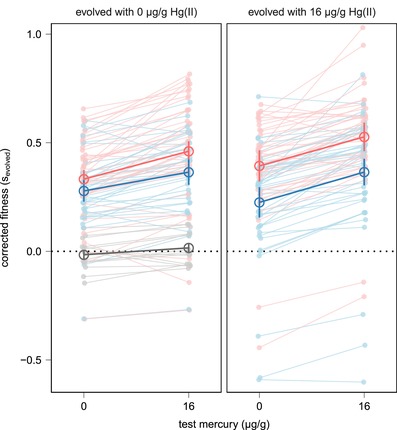
Clones evolved without *P. putida* (single‐species culture, red) achieved higher fitness gains than those evolved with *P. putida* (coculture, blue). Competition assays were conducted against plasmid‐containing control clones, with evolved plasmid‐containing clones from all plasmid‐containing populations. Six populations were tested for each combination of culture conditions (single‐species/coculture, evolved with/without Hg(II)), except where cocultured with 0 μg/g Hg(II) where only five populations maintained the plasmid. All fitness measurements were standardized by subtracting the mean fitness of the ancestral clone, indicated in gray. Six or seven evolved clones were tested from each population, and each clone was assessed under 0 μg/g Hg(II) and 16 μg/g Hg(II). Data from individual competitions are shown in lighter shades. Mean fitness for each population under each condition was calculated and used to calculate mean and standard error across all populations, shown in darker shades. We identified significant positive effects of test mercury (LMM χ²(1) = 62.111, *P* < 0.0001) and single‐species culture (χ²(1) = 4.934, *P* = 0.0263).

To examine the consequences of the different evolutionary treatments on adaptation of *P. fluorescens* SBW25, we isolated plasmid‐carrying clones from the end of the evolution experiment and measured their fitness relative to ancestral clones. Fitness was measured by direct competition in soil microcosms, and for each clone we measured fitness under 0 μg/g Hg(II) and under 16 μg/g Hg(II), to test whether evolution under the different conditions affected ability to cope with environmental mercury contamination. As expected, evolved clones generally showed enhanced fitness compared with the ancestor (i.e., relative fitness > 0) (Fig. 1), indicating adaptation to the soil environment. We detected a treatment‐specific effect of coculture: in general, clones which had evolved alongside *P. putida* achieved smaller fitness increases than those which had evolved in single‐species communities (LMM, effect of coculture χ²(1) = 4.934, *P* = 0.0263), suggesting that interspecific competition constrained abiotic adaptation. In addition, fitness increases were greatest when measured under 16 μg/g Hg(II) (effect of mercury χ²(1) = 62.111, *P* < 0.0001), regardless of mercury exposure during evolution, suggesting that this is likely to have been a by‐product of selection for growth in soil.

**Figure 2:**
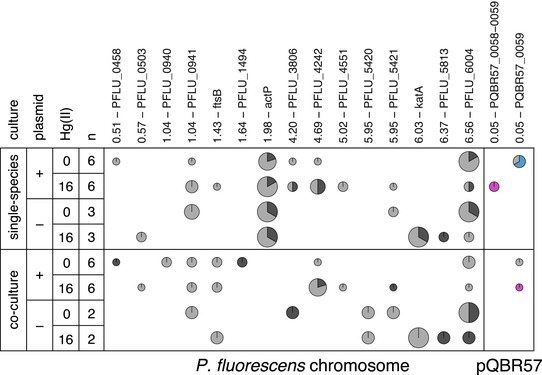
Parallel mutations detected in evolution experiment. Columns indicate loci with mutations occurring in >1 population, with the genomic location given in Mbp. Rows indicate different evolutionary treatments, with *n* indicating the number of populations within that treatment from which clones were subjected to whole‐genome resequencing and thus represented in the figure. A circle indicates mutation at that locus in that treatment in at least one population, with the size of the circle corresponding to the proportion of sequenced populations from that treatment in which mutations were detected. Pie sections are colored according to the types of mutation detected: mid‐gray = “moderate” impact SNV (substitutions); dark gray = “high” impact SNV (stop codons, indels); purple = Tn5042 insertion; blue = Tn6291 insertion. Data for *P. putida* are given in Figure S2. Genome sequencing data from Hall et al. (2017) is available on Dryad (https://doi.org/10.5061/dryad.6gf28).

To investigate further the differences between the single‐species and coculture evolved *P. fluorescens* clones, we compared the genome sequences of evolved clones (Hall et al. 2017). Parallel evolution, whereby the same genetic locus acquires mutations in multiple independently evolving lineages, is strong evidence for natural selection acting at this locus. Therefore, we examined locus‐level parallel mutations between lineages to identify differences in adaptive evolutionary trajectories between the treatments (Fig. 2 and Fig. S2). We used permutational MANOVA, which tests whether the same genes are more likely to be targeted within treatments than between treatments, to investigate the effects of treatment on genome evolution of both species. We detected significant effects of mercury treatment and coculture on *P. fluorescens* genome evolution (permutational MANOVA, effect of mercury *F*_1,21_ = 2.57, *P* = 0.0004; effect of coculture *F*_1,21_ = 3.42, *P* = 0.0001), but did not detect an effect of either treatment on *P. putida* genome evolution (effect of mercury *F*_1,21_ = 1.06, *P* = 0.33; effect of coculture *F*_1,21_ = 0.87, *P* = 0.71).

**Figure 3:**
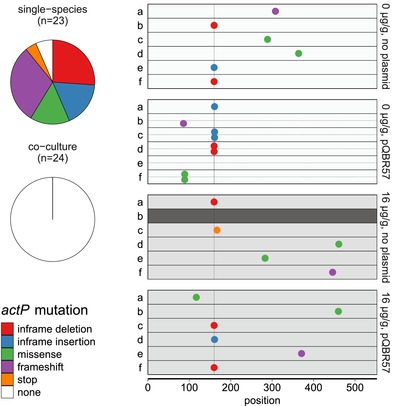
Evolved clones contained mutations in *actP*. (A) Pie chart showing types of *actP* mutation in single‐species and cocultured populations. All but one single‐species population contained the *actP* mutation, whereas it was found in none of the cocultured populations. (B) Chart indicating location of mutations in the amino acid sequence of 28 evolved clones. Mutations were not evenly distributed across ActP and tended to occur by expansion or contraction of a (TGG)_4_ trinucleotide repeat ∼160 codons from the start codon, indicated with a vertical line. Note that two clones were sequenced from five populations, separated by a horizontal dotted line. Genome sequencing data were supplemented by targeted sequencing of *actP* for single‐species plasmid‐free replicates b, e, and f, and cocultured plasmid‐free replicates b, c, e, and f.

We next tested whether specific parallel mutations were associated with the different treatments (Table S1 and Supporting Information Text). Only one *P. fluorescens* gene was identified following correction for multiple testing: *actP*, in which mutations were associated with single‐species culture (Fisher's exact test, Holm‐Bonferroni‐adjusted *P* = 0.000001). Mutations in *actP* drove the effect of coculture observed in the permutational MANOVA, as removing these data or randomizing them across populations abolished significance (effect of culture with *actP* mutations removed *F*_1,21_ = 1.3, *P* = 0.16). Supplementing our whole‐genome data with targeted sequencing of the *actP* gene of additional clones taken from five single species and eight cocultured populations, which were not whole‐genome sequenced, revealed *actP* mutations in 22 of 23 *P. fluorescens* single‐species populations (Fig. 3) (note that one plasmid‐free population went extinct under mercury selection). Mutation of *actP* was not associated with plasmid carriage, because mutations were present in both plasmid‐containing and plasmid‐free populations, nor was it associated with mercury treatment as it occurred in populations evolved with and without mercury. However, no *actP* mutations were detected in any of the cocultured *P. fluorescens* clones, potentially indicating a genetic basis for the fitness differences between the single species and cocultured clones.

There are two possible explanations for the lack of *actP* mutations in cocultured *P. fluorescens*. First, *P. fluorescens* population densities may be reduced in coculture, resulting in reduced mutational supply and lower evolutionary potential. Previously we did not detect an effect on the density of either *P. fluorescens* or *P. putida* grown in coculture compared with single‐species cultures over the course of a single transfer (Hall et al. 2016). Analyzing population density data from across the evolution experiment reveals that cocultured *P. fluorescens* did experience slightly reduced densities over the 65 transfers compared with single‐species culture (LMM, coculture‐transfer interaction χ²(1) = 10.01, *P* = 0.0016; Fig. S3). However, reduced population density in coculture is unlikely to explain the lack of *actP* mutations, because *actP* mutations arose in all the single‐species plasmid‐free mercury‐treated populations, where population density was much lower than that found in the cocultured plasmid‐bearing *P. fluorescens* populations (Fig. S3).

**Figure 4:**
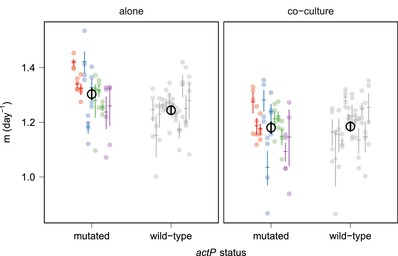
Clones with mutated *actP* achieved higher growth rates in single‐species cultures, but this effect was abolished in coculture. Evolved clones with either mutated *actP* or wild‐type *actP* were grown in single‐species culture (left) or coculture (right) and the growth rate (Malthusian constant *m*) calculated. Different colors represent different types of mutation, colored similarly to Figure 2. Red: inframe deletion; blue: inframe insertion; green: missense; purple: frameshift; gray: no mutation. Twenty‐three clones (11 actP mutants and 12 actP wild type) were tested in quadruplicate. Different points indicate independent technical replicates, bars indicate the mean and standard error for each clone, and the black circle and line indicate mean and standard error across clones. We identified a significant effect of *actP* status on growth in co‐culture compared with alone (LMM effect of *actP* status (χ²(1) = 12.034, *P* = 0.0005).

Alternatively, selection on *actP* may vary with community context, leading to fixation of *actP* mutations only in the single‐species treatments. To explore this possibility, we picked sequenced *P. fluorescens* clones with either mutated *actP* or wild‐type *actP* and grew them in soil microcosms either by themselves or alongside *P. putida*. Although each of these clones had at least one second‐site mutation (detailed in Table S2), none of these were confounded with the *actP* mutations, and presence or absence of mutations in *actP* was the only common factor between clones of each group. We measured *P. fluorescens* density after one growth cycle (4 days) (Fig. 4), and found that *actP* status had a significant effect on growth under the two experimental conditions (LMM, effect of actP status χ²(1) = 12.03, *P* = 0.0005). In the absence of *P. putida*, strains with mutated *actP* reached significantly higher densities than strains with the wild‐type *actP* (LMM, effect of actP status χ²(1) = 5.27, *P* = 0.022). However, this effect was abolished in the presence of *P. putida*, and there was no significant difference between strains with wild‐type *actP* and those with mutated *actP* (LMM, effect of actP status χ²(1) = 0.035, *P* = 0.85). This suggests that the net effect of *actP* disruption was beneficial, but only when *P. fluorescens* was grown alone, such that the fitness effects of losing *actP* varied with its community context.

Our analysis did not identify any mutations that were statistically associated with the coculture treatment. Intriguingly, however, further inspection of the sequencing data revealed the loss of a stop codon in a *P. fluorescens* pseudogene major facilitator superfamily (MFS) transporter, *PFLU_1494*, in two replicate cocultured populations. Although this event did not occur frequently enough to statistically associate with treatment, its appearance only in populations that had evolved in coculture with *P. putida* suggests that it could be linked to coculture adaptation. In one population, a A > T transversion in codon 224 of *PFLU_1494* replaced the TAG stop codon with a TTG leucine codon, and in another a T > C transition replaced the same stop codon with a CAG glutamine codon. In both cases, these mutations result in a predicted C‐terminal extension of 229 amino acids with six predicted transmembrane helices, resulting in a protein with >99% amino acid identity to previously annotated *Pseudomonas* MFS transporters (WP_046034536.1). This implies that evolution in coculture not only maintained one membrane transporter (ActP), but also potentially selected for reactivation of an additional, previously disrupted, transporter, albeit at a lower frequency.

## Discussion

We show that competitive species interactions can limit the evolutionary response to abiotic selection in a focal species. This occurred because the fitness benefits of abiotic adaptive mutations were negated in more complex communities. Although mutations in a nutrient‐scavenging transporter were highly beneficial and strongly selected in the absence of *P. putida*, these mutations did not increase growth rate and consequently were not selected in the presence of *P. putida*. This suggests that in more complex communities, *P. fluorescens* was under selection to retain a wider range of ecological functions, presumably due to stronger resource competition. Thus, interspecific competition altered the fitness landscape for *P. fluorescens* by effectively removing the dominant abiotic adaptive peak available to monoculture populations in soil. These findings contrast with previous studies of the same organism, where *P. fluorescens* evolutionary diversification of colony morphology was promoted by the presence of competitor species (Zhang et al. 2012; Jousset et al. 2016). Although colony morphologies are useful visual markers of diversity, how they translate to functional ecological traits such as resource use is often unknown. However, Joussett et al. (2016) did compare the metabolic phenotypes of the ancestral and evolved colony morphotypes. They found that the new colony morphotype, which was more likely to evolve with increasing diversity of competing strains, was more generalist in its metabolic function than the ancestor and had evolved to better exploit resources that were underused by the competitor community. This is consistent with our results in that both suggest that the presence of competitor species selects for genotypes of the focal species that possess a wider range of ecological functions than is favored in their absence. It is probable that, through more intense competition for the most productive resources, the presence of competing species acts to make the productivity of different resource niches more equitable for the focal species, which is likely to select for generalism (Jasmin and Kassen 2007).

In single‐species *P. fluorescens* cultures, mutations affecting *actP* arose in independently evolving populations, whereas cocultured clones consistently maintained the wild‐type *actP* allele. It is possible that some mutations, particularly in‐frame insertions and deletions, do not inactivate the protein (Sondek and Shortle 1990; Heinz et al. 1994) or are associated with change‐of‐function or gain‐of‐function (Ritz et al. 2001). However, in‐frame amino acid insertions and deletions can significantly disrupt protein structure and activity (Sondek and Shortle 1990; Vetter et al. 1996). Those observed here occurred in a conserved region predicted to form one of the ActP transmembrane helices (Jung 2002) and are predicted to be deleterious according to the PROVEAN tool (Choi et al. 2012). Furthermore, the observation that different types of mutation—frameshifts, missense, stop codon, and in‐frame insertions and deletions—appear in different populations suggests that selection in single‐species populations favors the same phenotype, namely loss‐of‐function.

ActP is a membrane‐spanning sodium‐solute symporter, the primary function of which is thought to be acquisition of acetate (Gimenez et al. 2003). Acetate is produced during sugar metabolism (Wolfe 2005) and can be present in plant root exudates (Badri and Vivanco 2009), and thus represents a potentially important carbon source in plant‐associated microbial communities. However, bacteria do not need *actP* for acetate acquisition because acetate can enter the cell passively by diffusion, and so ActP expression is likely to be useful only at very low acetate concentrations, that is, it is an acetate scavenger (Gimenez et al. 2003). Indeed, previous studies have shown that in *Escherichia coli* null mutants of *actP* grew about as well as wild‐type in well‐mixed laboratory media (Gimenez et al. 2003). Similarly, we were not able to quantify an effect of *actP* disruption on acetate growth phenotype (Fig. S4) in the evolved clones, suggesting that the phenotypic effect of loss of ActP may be subtle and/or only apparent in the soil environment. Although our data indicate that ActP was not beneficial in single‐species populations, its near universal loss suggests it was costly and therefore strongly selected against. By enhancing cell permeability, ActP has known pleiotropic effects, potentially increasing sensitivity to toxins that may be present in soil. For example, ActP mediates sensitivity to tellurite in the alphaproteobacterium *Rhodobacter capsulatus* (Borghese and Zannoni 2010) and to tellurite, zinc, and cadmium (the latter two of which can be common industrial pollutants of soil; Buchauer 1973; Tóth et al. 2016) in *E. coli* (Elías et al. 2015). However, we could not detect any effect on tellurite susceptibility in evolved clones (Fig. S5), suggesting a further unidentified cost of ActP expression. Indeed, these costs may be species specific, as we detected no mutations in *P. putida actP* (93% amino acid identical to *P. fluorescens actP*) either in single species or coculture.

In co‐culture, ActP function was retained, suggesting that acetate scavenging was a useful trait in competition. Studies of acetate metabolism in *E. coli* show that *actP* is expressed later in the growth cycle, as bacterial metabolism switches from a period of rapid growth in abundant nutrients to one of slower growth in more nutrient‐limited conditions (Wolfe 2005). *Escherichia coli* can evolve to specialize on acetate by switching to this resource earlier in the growth cycle, but this comes with the trade‐off of slower growth on other resources and thus is under frequency‐dependent selection (Friesen et al. 2004). A similar pattern may be at play in soil microcosms, where *P. fluorescens* SBW25 grows more slowly than *P. putida* KT2440, but reaches overall higher density (Hall et al. 2016). When grown alone, increased productivity for *P. fluorescens* in the early phase of the growth cycle may compensate for reduced nutrient‐scavenging efficiency later, but it is likely that in coculture, *P. fluorescens* was growing in an environment already colonized by *P. putida*. Under such conditions, competition for carbon sources is likely to be more intense, potentially enhancing the benefits of retaining acetate scavenging.

When organisms are in fluctuating environments, selection can alternately favor disruption and reactivation of genes encoding ecological functions (Lancaster and Masel 2009; Hammerschmidt et al. 2014). Differences in mutational target size mean that, in general, reversions are less likely to occur than disruptions, becoming increasingly unlikely over time due to epistasis between the disrupted locus and mutations at other sites (McCandlish et al. 2016). It is notable that most of the mutations in *actP* were through the expansion or contraction of an intragenic (TGG)_4_ trinucleotide repeat. Simple repeats of this kind undergo slipped‐strand mispairing and thus an increased rate of both mutation and reversion, which can function as a mutational mechanism for gene regulation (Moxon et al. 2006), for example, as with phase variation of the *Haemophilus influenzae* capsule (Power et al. 2009). Genes regulated by phase variation tend to encode cell‐surface molecules, including transporters (Theiss and Wise 1997), and have context‐dependent fitness effects (Moxon et al. 2006). It is possible that *actP* is also regulated in this way, given that its benefits seem to be affected by the community context: it is disrupted during periods of rapid expansion into new environments where resources are more plentiful, reverting to functionality where competitive species interactions favor efficient resource extraction.

The conservation of *actP* in *P. fluorescens* evolved in coculture suggests that interspecific competition selected for generalist resource use. Additionally, we observed parallel mutations to the stop codon of the *P. fluorescens* pseudogene *PFLU_1494* in two populations cocultured with *P. putida*. These mutations were predicted to restore a full‐length open‐reading frame encoding a putative MFS transporter. Although the precise function of *PFLU_1494* is unknown, it is tempting to speculate, based upon its homology to other known nutrient transporters, that this gene is also involved in resource acquisition, and that its reactivation may have allowed *P. fluorescens* to utilize a previously inaccessible resource. It is important to note, however, that unlike *actP*, *PFLU_1494* mutation was observed in too few replicate populations to be statistically associated with the coculture treatment. This could reflect a mutational bias, potentially due to a higher rate of mutation at the *actP* locus, or weaker selection for the stop‐codon reverting mutations at *PFLU_1494* (Lind et al. 2017).

Here, we have shown that species interactions can constrain abiotic adaptation of a focal species. The fitness benefits of abiotic adaptive mutations causing loss of a nutrient‐scavenging transporter were negated in the presence of a competitor species, which was selected for genotypes of the focal species that retained a wider range of ecological function. Interspecific competition altered the fitness landscape for the focal species, removing the dominant abiotic adaptive peak and rerouting evolution away from resource‐use specialization. These findings show that, unlike classic examples of character displacement for increased ecological specialization (Grant and Grant 2006), competitor species can also select for generalism. To better understand bacterial evolution in natural communities, it will be necessary to go beyond simple, single‐species experiments and adopt more complex, realistic environments and communities.

## CONFLICT OF INTEREST

The authors declare no conflict of interest.

Associate Editor: K. Lythgoe

## Supporting information

**Figure S1**. Experimental design.
**Figure S2**. Parallel mutations detected in evolution experiment.
**Figure S3**. Population densities at transfer of *P. fluorescens* from evolution experiment.
**Figure S4**. Growth in minimal acetate media compared with growth in minimal glycerol media.
**Figure S5**. Tellurite resistance of selected evolved clones.
**Table S1**. Parallel mutations detected in whole‐genome resequencing.
**Table S2**. Second‐site mutations in sequenced *P. fluorescens* clones.
**Table S3**. Data presented in Figure 1.
**Table S4**. Data presented in Figure 3.
**Table S5**. Data presented in Figure 4.
**Table S6**. Data presented in Figure S3.
**Table S7**. Data presented in Figure S4.
**Table S8**. Data presented in Figure S5A.
**Table S9**. Data presented in Figure S5B.
