# Supplementary material for: Competitive species interactions constrain abiotic adaptation in a bacterial soil community
Source: Evol Lett. 2018 Sep 25;2(6):580–9. doi: 10.1002/evl3.83 (PMC6292705; doi:10.1002/evl3.83)
Supplement: Supplementary file 2 — Figure S1. Experimental design. Figure S2. Parallel mutations detected in evolution experiment. Figure S3. Population densities at transfer of P. fluorescens from evolution experiment. Figure S4. Growth in minimal acetate media compared with growth in minimal glycerol media. Figure S5. Tellurite resistance of selected evolved clones. Table S1. Parallel mutations detected in whole‐genome resequencing. Table S2. Second‐site mutations in sequenced P. fluorescens clones. Table S3. Data presented in Figure 1. Table S4. Data presented in Figure 3. Table S5. Data presented in Figure 4. Table S6. Data presented in Figure S3. Table S7. Data presented in Figure S4. Table S8. Data presented in Figure S5A. Table S9. Data presented in Figure S5B. [file EVL3-2-580-s002.pdf]

Supporting Information for 'Competitive species interactions constrain abiotic adaptation in a bacterial soil community'

Authors: James P. J. Hall[1,2]*, Ellie Harrison[1], Michael A. Brockhurst[1]

Affiliations:

[1]Department of Animal and Plant Sciences, University of Sheffield, Western Bank, Sheffield, S10 2TN, United Kingdom

[2]Department of Biology, University of York, Wentworth Way, York, YO10 5DD, United Kingdom

*Correspondence to: j.p.hall@sheffield.ac.uk, Department of Animal and Plant Sciences, University of Sheffield, Western Bank, Sheffield, S10 2TN, United Kingdom. Tel: +44 (0) 141 222 0051.

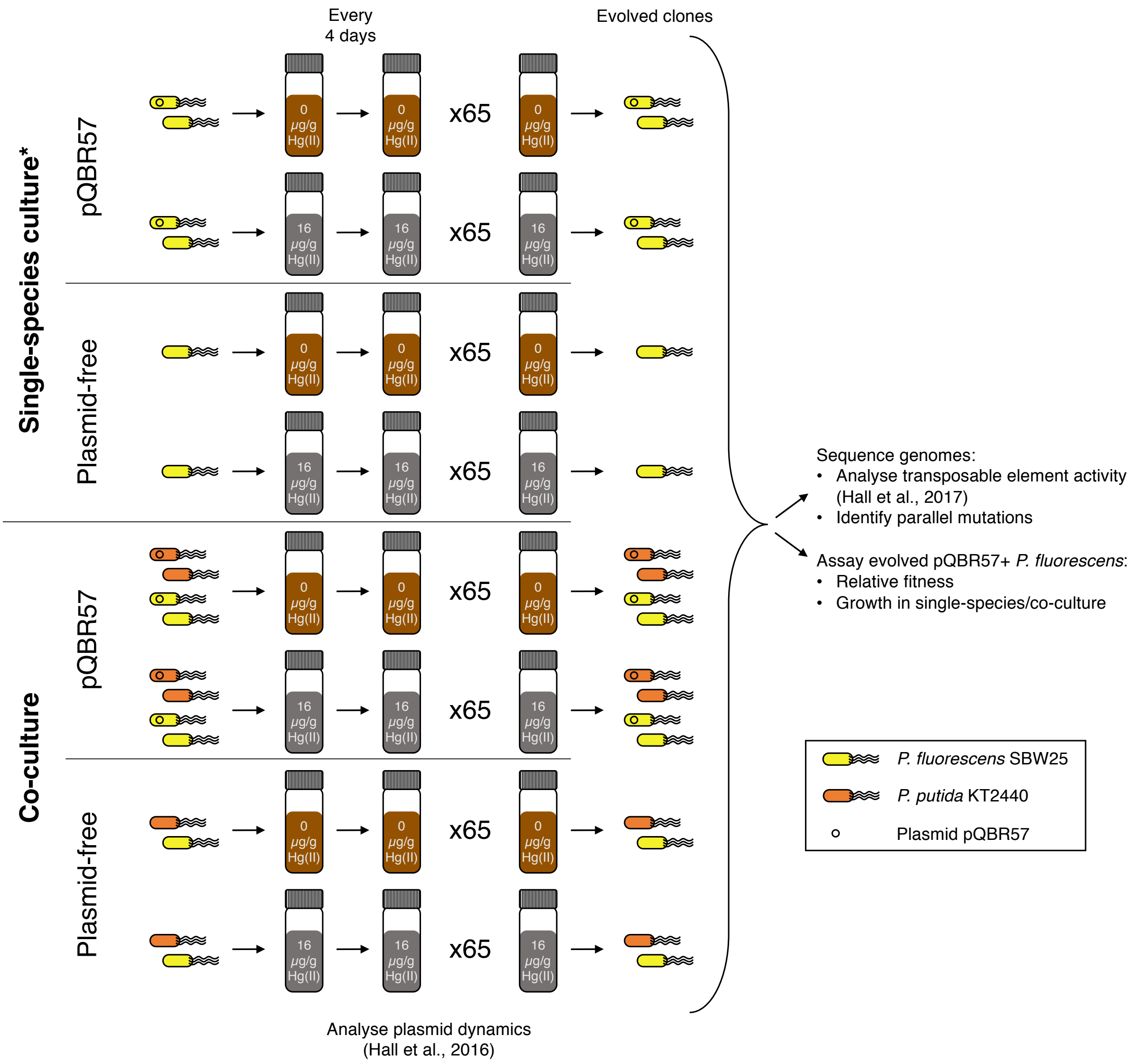

Figure S1. Experimental design. Cultures were established in soil microcosms in a full-factorial design with two levels of culture (single-species, co-culture), two levels of plasmid (plasmid-free, pQBR57), and two levels of mercury (0 µg/g Hg(II), 16 µg/g Hg(II)). Six replicates were established for each combination of treatments, only one is shown. Every four days samples were transferred into fresh media for a total of 65 growth cycles, after which clones were isolated and subjected to genomic and phenotypic analysis. *Note that similar single-species cultures were also established and run for *P. putida*.

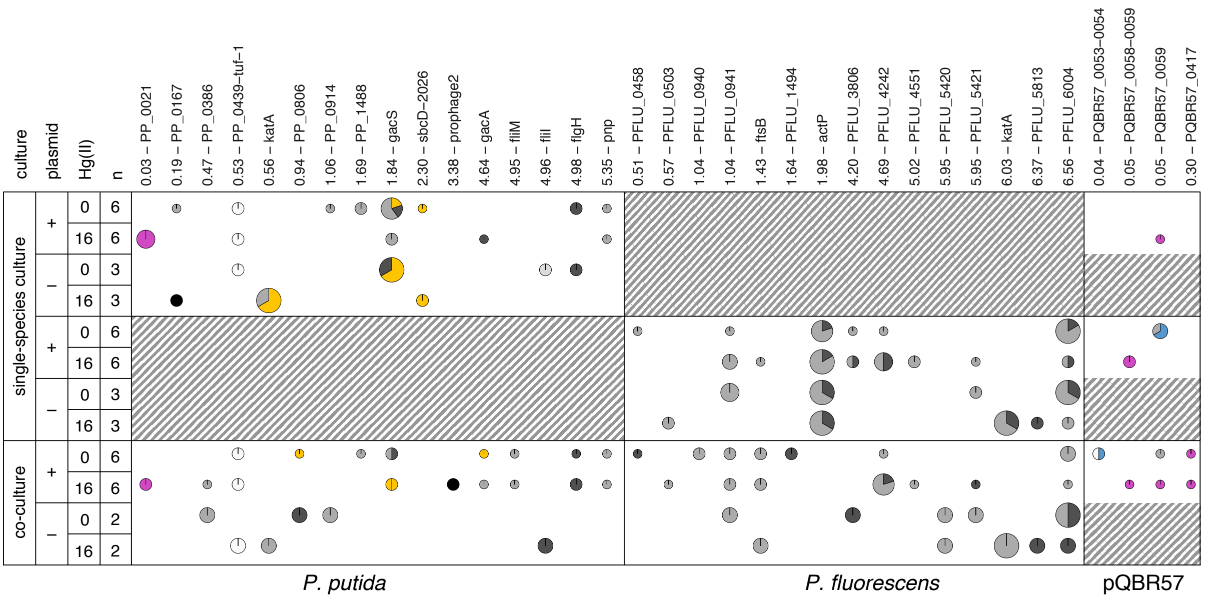

Figure S2. Parallel mutations detected in evolution experiment. As Figure 2, but including data for *P. putida*. Pie sections are coloured according to the types of mutation detected: white = ‘modifier’ (intergenic SNV); light-grey = ‘low’ impact SNV (synonymous mutation); mid-grey = ‘moderate’ impact SNV (substitutions, in-frame indels); dark grey = ‘high’ impact SNV (stop codons, frameshift); black = deletion; yellow = IS/Group 2 Intron insertion; purple = Tn5042 insertion; blue = Tn6291 insertion.

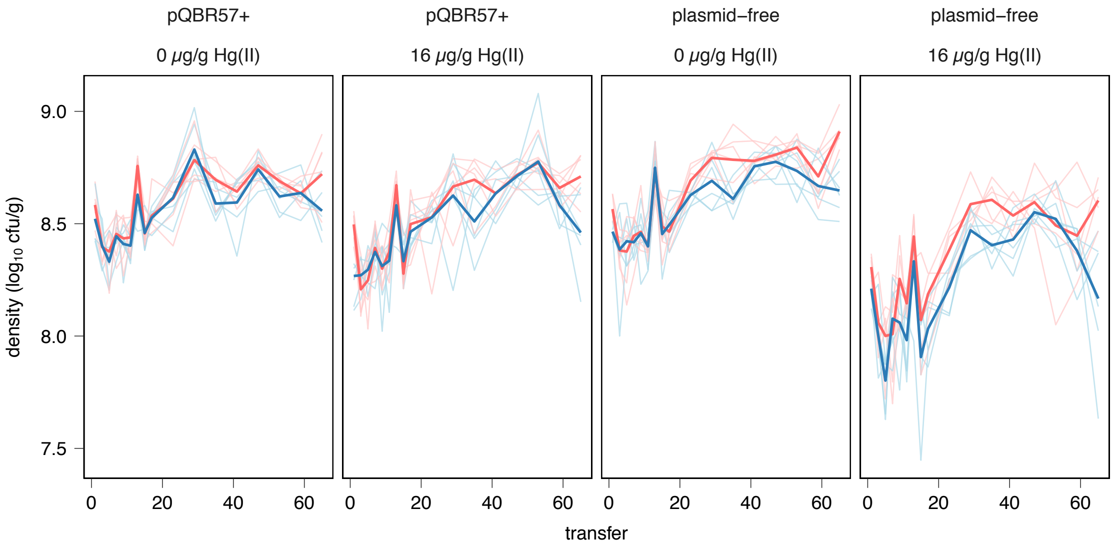

Figure S3. Population densities at transfer of *P. fluorescens* from evolution experiment. Note that some of this data is from (Hall *et al.* 2016) (Dryad Digital Repository doi:10.5061/dryad.488dk). Red indicates single-species culture and blue co-culture, while light coloured lines indicate independent populations (n=6 per treatment) and bold lines are the mean. We identified a negative effect of co-culture on densities over the course of the experiment (LMM co-culture:transfer interaction $\chi^2(1) = 10.01$, $p = 0.0016$), as well as a positive effect of plasmid carriage on populations grown in mercury over the course of the experiment (plasmid:mercury:transfer interaction $\chi^2(1) = 5.18$, $p = 0.023$).

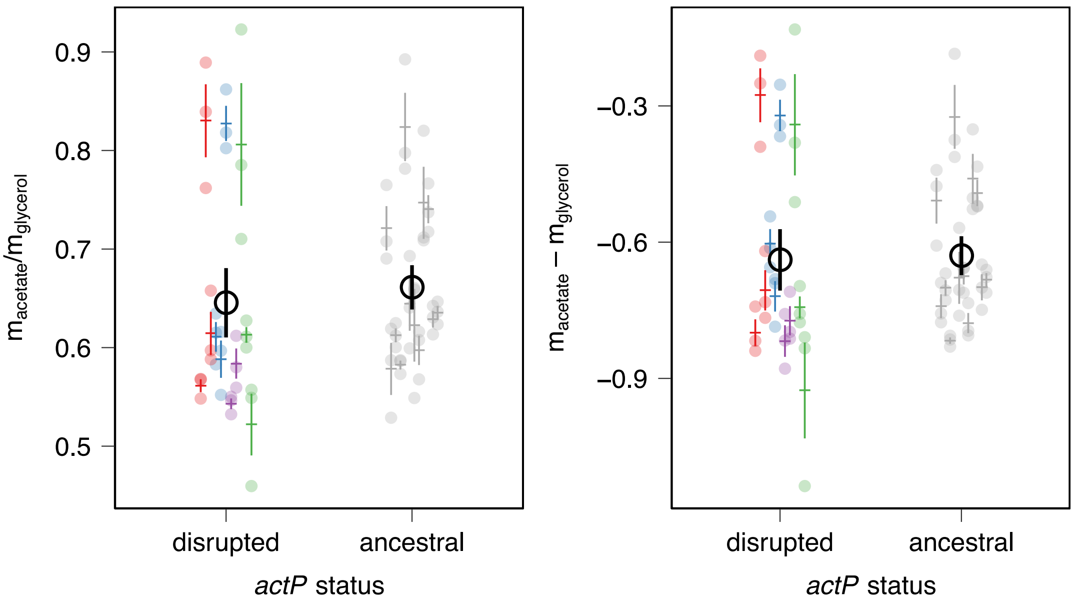

Figure S4. Growth in minimal acetate media compared with growth in minimal glycerol media. Growth rates in 1% glycerol (w/v) M9 minimal media ($m_{glycerol}$) and 0.3 mM acetate media ($m_{acetate}$) were measured for evolved sequenced clones. Points are coloured as in Figure 4. The left panel shows the ratio of Malthusian parameters whereas the right shows the difference in parameters: the former is more biologically intuitive (showing growth in acetate as a proportion of growth in glycerol) whereas the latter is more mathematically meaningful (since ratios on a linear scale correspond to subtractions on a logarithmic scale). Regardless of measure used, no significant difference was detected between clones with ancestral or disrupted *actP* loci (LMM effect of *actP* status on ratio $\chi^2(1) = 0.75931$, $p = 0.3835$; effect on difference $\chi^2(1) = 0.22186$, $p = 0.6376$).

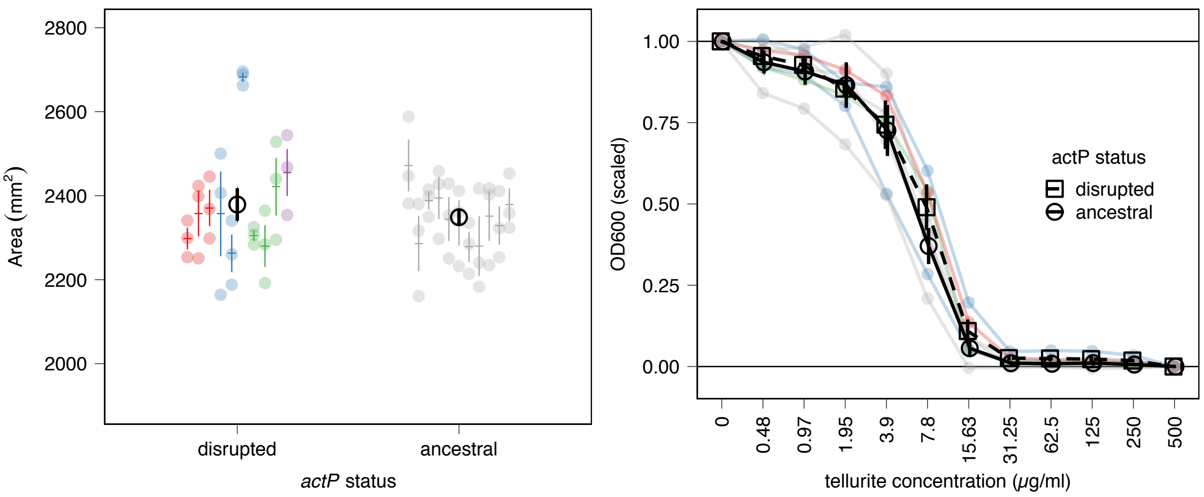

Figure S5. Tellurite resistance of selected evolved clones. Left: Zones of inhibition were measured in triplicate for clones with disrupted (n = 10) or undisrupted (n = 11) *actP* genes. Points are coloured as in Figure 4. No significant difference between the two groups was detected (LMM effect of *actP* status $\chi^2(1) = 0.3741$, $p = 0.5408$). Right: OD_{600} of cultures grown for 48 h in KB supplemented with various concentrations of K_2TeO_3, scaled as a proportion of growth in 0 µg/ml K_2TeO_3. Triplicate cultures were grown for clones with disrupted or undisrupted *actP* genes and the mean for each clone is indicated (n = 4 for each). Points are coloured as in Figure 4, with black circles and squares indicating the mean across ancestral and disrupted *actP* variants respectively. No significant difference between ancestral and disrupted *actP* was detected (LMM effect of *actP* status $\chi^2(1) = 0.60427$, $p = 0.437$).

	culture	single-species culture				co-culture				total	p (culture)	p (mercury)	p (plasmid)	p<alpha	p<alpha (adj)	notes
	plasmid	pQBR57		plasmid-		pQBR57		plasmid-								
	Hg(II) (µg/g)	0	16	0	16	0	16	0	16							
species		n=6	n=6	n=3	n=3	n=6	n=6	n=2	n=2							
P. putida	*flgH*	2	0	1	0	1	2	0	0	6	1.0000	0.6562	0.6445			flagellum operon (flagellar L-ring protein precursor); upstream region also targeted (1 clone)
P. putida	*fliI*	0	0	1	0	0	0	0	1	2	1.0000	1.0000	0.0802			flagellum operon (flagellum-specific ATP synthase)
P. putida	*fliM*	0	0	0	0	1	1	0	0	2	0.2139	1.0000	1.0000			flagellum operon (flagellar motor switch protein)
P. putida	*gacA*	0	1	0	0	1	1	0	0	3	0.5909	1.0000	0.5388			gacA/S
P. putida	*gacS*	5	2	3	0	2	2	0	0	14	0.0921	0.0799	0.4674			gacA/S
P. putida	*katA*	0	0	0	3	0	0	0	1	4	0.6041	0.1026	**0.0045**	*		catalase
P. putida	*pnp*	1	1	0	0	1	1	0	0	4	1.0000	1.0000	0.2958			polyribonucleotide nucleotidyltransferase
P. putida	*PP_0021*	0	4	0	0	0	2	0	0	6	0.6602	**0.0184**	0.1478	*		hypothetical; targeted by Tn5042; upstream region also targeted (1 clone)
P. putida	*PP_0167*	1	0	0	1	0	0	0	0	2	0.4866	1.0000	0.5080			targeted by SNV and by deletion
P. putida	*PP_0386*	0	0	0	0	0	1	1	0	2	0.2139	1.0000	0.5080			sensory box protein
P. putida	*PP_0439-tuf-1*	2	2	1	0	2	2	0	1	10	1.0000	1.0000	0.6833			intergenic; Tu elongation factor
P. putida	*PP_0806*	0	0	0	0	1	0	1	0	2	0.2139	0.4848	0.5080			large (6310aa) surface adhesion protein
P. putida	*PP_0914*	1	0	0	0	0	0	1	0	2	1.0000	0.4848	0.5080			GGDEF domain protein
P. putida	*PP_1488*	2	0	0	0	1	0	0	0	3	1.0000	0.2273	0.5388			methyl-accepting chemotaxis transducer
P. putida	*prophage2*	0	0	0	0	0	2	0	0	2	0.2139	0.4848	1.0000			prophage 2
P. putida	*sbcD-2026*	1	0	0	1	0	0	0	0	2	0.4866	1.0000	0.5080			exonuclease SbcD
P. fluorescens	*actP*	5	6	3	3	0	0	0	0	17	**0.0000**	1.0000	0.7080	*	*	acetate scavenger
P. fluorescens	*ftsB*	0	1	0	0	2	2	0	1	6	0.0782	0.6562	0.6445			cell division protein
P. fluorescens	*katA*	0	0	0	3	0	0	0	2	5	1.0000	**0.0445**	**0.0009**	*		catalase
P. fluorescens	*PFLU_0458*	1	0	0	0	1	0	0	0	2	1.0000	0.4848	1.0000			conserved GGDEF/EAL domain protein
P. fluorescens	*PFLU_0503*	0	0	0	1	0	1	0	0	2	1.0000	0.4848	0.5080			putative membrane protein; upstream region also targeted
P. fluorescens	*PFLU_0940*	0	0	0	0	2	0	0	0	2	0.2139	0.4848	1.0000			putative cell division protein
P. fluorescens	*PFLU_0941*	1	3	2	0	2	2	1	0	11	1.0000	1.0000	1.0000			putative peptidoglycan glycosyltransferase; cell-division related
P. fluorescens	*PFLU_1494*	0	0	0	0	2	0	0	0	2	0.2139	0.4848	1.0000			transport-related membrane pseudogene; stop lost in evolved genes
P. fluorescens	*PFLU_3806*	1	2	0	0	0	0	1	0	4	0.6041	1.0000	1.0000			ATP-dependent clp protease adaptor protein
P. fluorescens	*PFLU_4242*	1	4	0	0	1	5	0	0	11	0.7166	**0.0255**	**0.0135**	*		DUF262 domain of unknown function
P. fluorescens	*PFLU_4551*	0	2	0	0	0	1	0	0	3	1.0000	0.2273	0.5388			putative aerotaxis sensor receptor
P. fluorescens	*PFLU_5420*	0	0	0	0	0	0	1	1	2	0.2139	1.0000	0.0802			putative lipoprotein
P. fluorescens	*PFLU_5421*	0	1	1	0	0	1	1	0	4	1.0000	1.0000	0.5636			putative peptidoglycan-related
P. fluorescens	*PFLU_5813*	0	0	0	1	0	0	0	1	2	1.0000	0.4848	0.0802			putative heavy metal ABC transport system
P. fluorescens	*PFLU_6004*	6	2	3	1	3	1	2	1	19	0.2998	**0.0049**	0.4513	*		conserved hypothetical (Interpro: aminoacyl-tRNA editing activity)
pQBR57	*PQBR57_0053-0054*	0	0	0	0	2	0	0	0	2						upstream of parA
pQBR57	*PQBR57_0058-0059*	0	2	0	0	0	1	0	0	3						lambda-repressor-like
pQBR57	*PQBR57_0059*	3	1	0	0	1	1	0	0	6						lambda-repressor-like
pQBR57	*PQBR57_0417*	0	0	0	0	1	1	0	0	2						NAD(P) binding domain

Table S1. Parallel mutations detected in whole-genome resequencing. Parallel mutations (n=35) were identified as loci mutated in clones from >1 population. To identify mutations associated with the different treatments, parallel chromosomal mutations occurring in >1 population from one treatment and not in the other level of that treatment (n=23) were subjected to sequential Bonferroni-corrected Fisher's Exact Test for association with each treatment (m=23x3 tests). A significant result before adjustment for multiple testing is indicated by an asterisk, an additional asterisk in the ‘p < alpha (adj)’ indicates significance after multiple testing. For comparison, Fisher's Exact Test results are provided for all parallel chromosomal loci. Loci of interest are discussed in Supplementary Text.

sample number	co-culture/single-species	evolved mercury	evolutionary replicate	used for tellurite MIC	0.00 - PQBR57_0003	0.04 - PQBR57_0053-0054	0.05 - PQBR57_0058-0059	0.05 - PQBR57_0059	0.15 - PQBR57_0173-0174	0.16 - PQBR57_0189*	0.16 - PQBR57_0190-0191*	0.20 - PQBR57_0227-0228	0.20 - PQBR57_0228	0.20 - PQBR57_0228-0229	0.25 - PQBR57_0316-0317	0.25 - PQBR57_0327	0.25 - PQBR57_0330-0331	0.25 - PQBR57_0332	0.30 - PQBR57_0417	0.02 - PFLU_0020	0.33 - PFLU_0297-0299	0.33 - PFLU_0299-wssA	0.54 - PFLU_0477	0.57 - PFLU_0503	0.57 - PFLU_0503-0504	1.04 - PFLU_0940	1.04 - PFLU_0941	1.06 - PFLU_0950	1.08 - PFLU_0971	1.32 - PFLU_1195	1.43 - ftsB	1.64 - PFLU_1494	1.96 - glxR	1.98 - actP	2.06 - PFLU_1884	2.06 - PFLU_1887	2.06 - PFLU_1888	2.08 - eno	2.69 - PFLU_2479	2.70 - PFLU_2487	3.32 - PFLU_3046	3.33 - PFLU_3050-3051.1	3.33 - PFLU_3051.1-3051.2	3.60 - PFLU_3249	3.81 - PFLU_3436-3437	4.20 - PFLU_3806	4.32 - PFLU_3920	4.68 - PFLU_4242	4.88 - PFLU_4418-4419	4.90 - PFLU_4439	5.02 - PFLU_4551	5.80 - PFLU_5285	5.83 - PFLU_5309	5.95 - PFLU_5421	6.15 - PFLU_5616	6.56 - PFLU_6004
49	co	0	b	Y																																																				
73	co	0	c	Y																																																				
16	co	0	d																																																					
56	co	0	e																																																					
80	co	0	f																																																					
13	co	16	a	Y																																																				
53	co	16	b																																																					
77	co	16	c	Y																																																				
20	co	16	d																																																					
59	co	16	e																																																					
84	co	16	f																																																					
42	ss	0	e																																																					
1	ss	0	a	Y																																																				
39	ss	0	b																																																					
62	ss	0	c	Y																																																				
3	ss	0	d																																																					
65	ss	0	f																																																					
2	ss	16	a	Y																																																				
41	ss	16	b																																																					
64	ss	16	c	Y																																																				
5	ss	16	d																																																					
44	ss	16	e																																																					
67	ss	16	f																																																					

Table S2. Second-site mutations in sequenced *P. fluorescens* clones. Filled cells indicate a mutation was detected in that clone, with fill colour corresponding to mutation type: speckled white = 'modifier' (intergenic SNV); light-grey = 'low' impact SNV (synonymous mutation); mid-grey = 'moderate' impact SNV (substitutions, in-frame indels); dark grey = 'high' impact SNV (stop codons, frameshift); black = deletion; hatched purple = Tn5042 insertion; hatched green = Tn4652 insertion; hatched orange = Tn6290 insertion; hatched blue = Tn6291 insertion. For the *actP* insertions, the nature of the mutation is given by the colour, corresponding to Figure 4: red = inframe deletion; blue = inframe insertion; green = missense; purple = frameshift. Mobile genetic element insertions in PQBR57_0189 and PQBR57_0190-0191, indicated by a *, did not occur independently (i.e. they were present in ancestral clones) and thus not presented in Figure 2 or Supplementary Table 1.

Table S3. Data presented in Figure 1.

Table S4. Data presented in Figure 3.

Table S5. Data presented in Figure 4.

Table S6. Data presented in Figure S3.

Table S7. Data presented in Figure S4.

Table S8. Data presented in Figure S5A.

Table S9. Data presented in Figure S5B.

Tables S3-S9 are provided as an Excel workbook.

Supplementary Text: Discussion of parallel mutations

We detected 35 loci which were mutated in >1 population (Supplementary Table 1). Of these, only *actP* from *Pseudomonas fluorescens* SBW25 was significantly associated with an experimental treatment following correction for multiple testing. Three other genes were identified as non-significant following correction for multiple testing: *katA* and *PFLU_4242* (also from *P. fluorescens* SBW25), and *katA* and *PP_0021* (from *P. putida* KT2440). These, as well as other parallel mutations of interest, are discussed here.

actP acetate scavenging transporter (*P. fluorescens*). Discussed in the main text.

katA catalase (*P. fluorescens* and *P. putida*). Mutations appeared only in populations grown without pQBR57 and only in populations grown in mercury, and appeared in all but one clone (*P. putida)* sequenced from this treatment. Mutations are therefore associated with populations without specific mercury resistance growing in mercury. Hg(II) is detoxified by reduction to Hg(0), which can occur through the activity of the *merA* reductase (which occurs in the plasmid treatments) or can occur abiotically (Barkay *et al.* 2003). Catalase promotes the opposite reaction, oxidizing elemental Hg(0) to toxic Hg(II) (Smith *et al.* 1998) (although at a lower rate than *merA*-mediated reduction (Barkay *et al.* 2003)). Disruption of catalase will therefore favour a less toxic Hg redox state, likely to be particularly important in populations without *merA*.

PFLU_4242 hypothetical protein (*P. fluorescens*). Mutations only appeared in plasmid-containing treatments and were more frequent in the presence of mercury. Possibly involved in amelioration of plasmid cost.

PP_0021 hypothetical protein (*P. putida*). This gene was only mutated by Tn5042 mercury resistance transposon insertion, occurring in plasmid-containing populations in the presence of mercury. It likely represents an insertion hotspot (Oliveira *et al.* 2017).

gacA/S global regulator (*P. putida*). The *gacA/S* regulator was frequently disrupted in *P. putida* (but never in *P. fluorescens*) across treatments, however unlike for *P. fluorescens* (cf. Harrison *et al.* 2015), *P. putida gacA/S* disruption is unlikely to be connected with plasmid amelioration since mutations also arose in plasmid-free treatments.

PFLU_0940 putative cell division protein*,* ***PFLU_0941*** putative peptidoglycan glycosyltransferase, ***PFLU_5421*** peptidoglycan-related, ***ftsB*** cell division protein (*P. fluorescens*). Genes putatively involved in cell division. Mutations appeared in all treatments, and thus are likely to be associated with media and/or transfer regimen. Similarly, ***PFLU_6004*** hypothetical protein was frequently mutated across treatments and likely to be a general adaptation.

flgH flagellar L-ring precursor, ***fliH*** flagellar assembly protein H, ***fliI*** flagellum-specific ATP synthase, ***fliM*** flagellar motor switch protein (*P. putida*). Genes involved in flagellum synthesis. Mutations appeared in all treatments and are thus likely to be a general adaptation to the evolution experiment. A previous study with *Salmonella* calculated that flagellar operation can consume ~4.5% of the cell's energy expenditure, and thus disrupting this operon could be adaptive under conditions where the flagellum is not beneficial (Koskiniemi *et al.* 2012).

PQBR57_0059 lambda-repressor-like protein (pQBR57). Plasmid locus disrupted in both species. Possibly involved in amelioration of plasmid cost.

Supplementary Text References

1.Barkay, T., Miller, S.M. & Summers, A.O. (2003). Bacterial mercury resistance from atoms to ecosystems. *FEMS Microbiol Rev*, 27, 355–384.

2.Harrison, E., Guymer, D., Spiers, A.J., Paterson, S. & Brockhurst, M.A. (2015). Parallel compensatory evolution stabilizes plasmids across the parasitism-mutualism continuum. *Curr. Biol.*, 25, 2034–2039.

3.Koskiniemi, S., Sun, S., Berg, O.G. & Andersson, D.I. (2012). Selection-Driven Gene Loss in Bacteria. *PLoS Genet*, 8.

4.Oliveira, P.H., Touchon, M., Cury, J.Eduardo P. C. Rocha. (2017). The chromosomal organization of horizontal gene transfer in bacteria. *Nature Communications*, 8:841.

5.Smith, T., Pitts, K., McGarvey, J.A. & Summers, A.O. (1998). Bacterial oxidation of mercury metal vapor, Hg(0). *Applied and Environmental Microbiology*, 64, 1328–1332.
